
JOURNAL INFORMATION
==============================
NLM Title Abbreviation: Gastroenterol Rep (Oxf)
Journal ID: Gastroenterol Rep (Oxf)
NLM Journal ID: 101620508
Journal ID: gastro

Gastroenterology Report

EISSN: 2052-0034
Publisher: Oxford University Press

ARTICLE INFORMATION
==============================
PMCID: PMC5952936
DOI: 10.1093/gastro/goy015
Article ID: goy015
Article version: 1
Subjects: Chinese Abstracts

Chinese Abstracts – Volume 6 Issue 2*

Print publication date: 2018 May
Electronic publication date: 2018 May 14
Volume: 6
Issue: 2
First page: C1
Last page: C3
Copyright: © The Author(s) 2018. Published by Oxford University Press and Sixth Affiliated Hospital of Sun Yat-Sen University
Copyright year: 2018
License: This is an Open Access article distributed under the terms of the Creative Commons Attribution License (http://creativecommons.org/licenses/by/4.0/), which permits unrestricted reuse, distribution, and reproduction in any medium, provided the original work is properly cited.
License URL: https://creativecommons.org/licenses/by/4.0/

Page count: 3

==============================
OXFORD

*Gastroenterology Report*, 2018, 1–3

doi: 10.1093/gastro/goy015

### Chinese Abstracts – Volume 6 Issue 2*

### Review（综述）

### Role of endoscopy in inflammatory bowel disease
### 内镜在炎症性肠病中的作用

[摘要]　克罗恩病（CD）和溃疡性结肠炎（UC）是炎症性肠病（IBD）两种最常见的类型。回结肠镜加活检被认为是IBD诊断的金标准。CD与UC的鉴别诊断是非常重要的，因为它们的治疗策略及预后是有所不同的。然而，约有15%的IBD患者由于未能明确诊断为CD或UC而错误地归入了不定型肠炎。内镜在IBD中的作用受到了越来越多的关注。例如，一些新的影像技术有助于CD与UC的内镜下鉴别诊断。内镜球囊扩张和内镜下支架放置已经CD狭窄治疗的重要组成部分。在CD患者的肛瘘检查中，内镜超声也被认为较磁共振更加准确。此外，色素内镜较普通白光肠镜能更早期发现不典型增生。因此，内镜已经成为IBD诊断、治疗以及并发症处理的基石。近年来，内镜在IBD中的作用不断提升，从小肠成像到内镜球囊扩张以及色素内镜对不典型增生的监测。本文我们讨论了内镜用于IBD诊断、治疗和并发症处理的最新证据。

关键词：炎症性肠病；小肠成像；治疗内镜；色素内镜；内镜球囊扩张

### Early detection of ulcerative colitis-associated colorectal cancer
### 溃疡性结肠炎相关结直肠癌的早期发现

[摘要]　结肠炎相关结直肠癌（CACC）是炎症性肠病（IBD）尤其是溃疡性结肠炎（UC）最严重的并发症之一，占所有IBD患者死亡原因的15%。由于相较于散发性结直甩接口，CACC预后不佳，死亡率较高，因此，早期发现是提高疗效的关键。结肠镜被推荐作为监测的首选方法，一些高级内镜成像技术也不断涌现。此外，最近不少研究都致力于采用不同的生物样本找到临床相关的监测标志物，这些标志物未来可能成为高效的筛查工具。所以说，对于病程较长的UC患者的癌变监测，最理想的监测方法目前仍存在争议。本文对早期发现CACC的相关研究进展进行了全面描述和总结。

关键词：溃疡性结肠炎；结肠炎相关结直肠癌；内镜；生物标志物；监测

### Original article（论著）

### Value of routine stool testing for pathogenic bacteria in the evaluation of symptomatic patients with ileal pouches
### 常规粪便病原菌培养对有症状的回肠储袋患者的评估价值

[摘要]　背景：对于有症状的回肠储袋患者，临床上经常会进行粪便培养以明确肠道病原菌。本研究旨在探究常规粪便培养对于有症状的回肠储袋患者的评估价值，以及找到肠道病原菌对于这些患者的临床意义。

方法：检索我们储袋数据库中2002－2015年间的2283例病例资料，筛选出其中有症状（提示储袋炎）且进行了粪便培养的病例纳入研究。将粪便培养结果阳性的患者作为研究组，并按1:4的比例进行匹配，选取粪便培养结果阴性的有症状患者作为对照。比较两组患者粪便培养1个月和3个月后的抗生素治疗反应、复发率和入院率。

结果：共计643例（28%）患者出现储袋炎症状且进行了粪便培养，其中只有1.7%（11/643）的患者培养结果阳性。空肠弯曲杆菌（45%）是最常见的致病菌，其次是气单胞菌（36%）。不抽烟和近3个月未使用任何抗生素的患者，其培养结果阳性率更高（$P < 0.001$和$P = 0.023$）。具有病原菌（培养结果阳性）的患者急性肾衰风险（27.3% vs. 4.5%，$P = 0.049$）、3个月内住院率（36.4% vs. 6.8%，$P = 0.009$）以及病死率（18.2% vs. 0%，$P = 0.040$）均显著高于对照组。然而，对于培养结果阳性的患者，基于病原菌的针对性治疗后，其临床疗效与对照组相比并无显著改善。

结论：我们发现对有症状的储袋患者进行粪便培养，其病原菌检出率极低，而且针对病原菌的治疗对储袋炎的疾病进程影响甚微。因此，对于这些患者是否有必要进行常规粪便培养需进一步研究。

关键词：储袋炎；回肠储袋；肠道病原菌；粪便培养

*This article abstract was originally published in English. This translation has not been verified and should not be relied upon—it is provided for reference purposes only. The Publishers have not checked this translation and accept no liability for completeness or accuracy of this translation or the use of this translation for whatever purpose. This translation may be incomplete and inaccurate in whole or in part. If you need to rely upon a translation of this abstract, a professional human translator should be engaged to supply an accurate translation of the original English. When referencing articles from this journal, please always refer to the original English version, rather than a translated equivalent.
本文摘要最初是以英文发表。中文摘译未获认证，谨供参考。出版社没有对中文摘译进行核验，不负责译文的完整性和准确性，也不会将其用以其他目的。中文摘译可能会出现部分不完整或不准确的情况。如果你需要准确获悉摘要，最好能请一位专业译者针对英文原文进行翻译。阅读杂志文章，请参考英文原文，不推荐中文翻译。

### Outcomes and prognostic factors of fecal microbiota transplantation in patients with slow transit constipation: results from a prospective study with long-term follow-up

### 粪菌移植应用于慢传输型便秘的临床疗效及疗效影响因素：一项前瞻性研究的长期随访结果

[摘要] 背景和目的 肠道菌群参与结肠动力的调控，菌群失调是便秘是病因之一。粪菌移植被证实可以恢复肠道平衡。本研究旨在评估粪菌移植治疗慢传输型便秘（STC）的临床疗效和预后影响因素。

方法：52例STC患者接受标准的粪菌移植入组研究，对其进行至少6个月的随访。记录入组患者的排便习惯、结肠传输时间、便秘相关的症状（PAC-SYM评分）、生活质量（PAC-QOL评分）、治疗满意度和不良反应。主要疗效指标是平均每周完全自主排便≥3次的患者比例。

结果：治疗后34周、912周、2124周，每周完全自主排便≥3次患者的比例分别为50.0%、38.5%和32.7%，均较治疗前显著提高（均P < 0.01）。其他排便情况、结肠传输时间、便秘相关症状和生活质量方面，治疗后亦有显著改善；但这些短期改善在治疗后12周和24周逐渐减小。治疗前排便不尽是唯一的疗效影响因素。治疗和随访期间未发现任何治疗相关的不良反应。

结论：本研究显示粪菌移植治疗STC安全有效，但远期疗效有所降低。治疗前排便不尽感较少甚至没有的患者接受粪菌移植的治疗效果更佳。

关键词：粪菌移植；慢传输型便秘；肠道菌群；结肠动力；预后因素

### The impact of surgeon choices on costs associated with uncomplicated minimally invasive colectomy: you are not as important as you think

### 外科医生对微创结肠切除手术医疗费用的影响：你并没有您想像中的那么重要

[摘要] 背景：在美国，民众对持续增长的医疗费用的关注度越来越高。对于简单的微创结肠切除手术（结肠肠段切除术且术后未出现重要并发症）的治疗费用，外科医生本身是否会产生影响目前尚无数据报道。

方法：分析我院医疗成本数据库中近一年来行择期微创结肠肠段切除手术患者的手术及术后治疗相关的直接医疗费用。

结果：共计111例患者纳入研究，其中18例行机器人手术。微创结肠切除手术的平均直接费用是5536美元，机器人手术比腹腔镜手术高53%（7806美元 vs. 5096美元，P < 0.001）。3名外科医生所施行的腹腔镜手术，其总体医疗费用分别为5099美元、5108美元和5055美元（P = 0.987）；其中，手术室费用分别是1236美元、1105美元和1030美元（P = 0.067），标准差为328美元（占总费用的6.4%）。

结论：对于微创结肠切除手术患者，就治于不同外科医生所产生的总体医疗费用并无显著差异，无论外科医生资历高低、经验水平和手术技术。总体医疗费用在同一机构是相对固定的，外科医生个体对其影响甚微。

关键词：医疗费用；微创结肠切除术；腹腔镜；外科医生

### Perioperative blood transfusion is associated with post-operative infectious complications in patients with Crohn's disease

### 围手术期输血增加克罗恩病术后感染性并发症发生率

[摘要] 背景：我们中心的数据显示，输血会增加克罗病术后并发症。本研究旨在基于美国外科医师协会的国家手术质量提升计划（ACS NSQIP）这个大数据平台，验证围手术期输血与克罗恩病患者术后感染性并发症的关系。

方法：从NSQIP数据库中提取2005－2013年间的克罗恩病手术病例的临床资料。参照ACS NSQIP指南对相关的临床变量进行定义和分层。主要结局指标是感染性并发症，包括外科手术部位感染（SSI，包括切口浅部感染、切口深部感染、器官/腔隙感染）、肺炎、泌尿系感染、脓毒血症及中毒性休克。对术后感染的危险因素进行多因素分析。

结果：10 100例病例纳入研究，其中611例（6.0%）接受了围手术期输血。输血患者年龄相对更大，体质量更轻，且功能依赖（即生活不能完全自理，一定程度上需要依赖他人）的比例相对更高。与未输血患者相比，输血患者术后感染性并发症发生率较高，包括切口浅部感染（10.8% vs. 7.4%，P = 0.002），切口深部感染（3.3% vs. 1.6%，P = 0.003），器官/腔隙感染（14.2% vs. 5.4%，P < 0.001），肺炎（3.8% vs. 1.3%，P < 0.001），泌尿系感染（3.9% vs. 2.2%，P = 0.006），脓毒血症（11.5% vs. 4.5%，P < 0.001），中毒性休克（3.1% vs. 0.8%，P < 0.001）。多因素分析结果显示，术中和/或术后输血是术后感染性并发症的独立危险因素（OR = 2.2，95% CI：1.8－2.7，P < 0.001），每增加一个单位的红细胞输入，术后感染风险增加30%（OR = 1.3，95% CI：1.2－1.5）。其他独立危险因素还包括吸烟、心脏病、糖尿病、高血压和服用类固醇类药物。但术前输血并不是术后感染的危险因素。

结论：术中和/或术后输血会增加克罗恩病术后感染性并发症的发生风险，但术前输血对此并无影响。因此，临床上需合理选择输血时机并仔细权衡输血的风险与获益。

关键词：输血；克罗恩病；感染性并发症；外科疗效

### Fluoroscopic gastric peroral endoscopic pyloromyotomy (G-POEM) in patients with a failed gastric electrical stimulator

### 透视引导的内镜下幽门肌切开术（G-POEM）治疗胃电刺激无效的顽固性胃瘫

[摘要] 背景：胃电刺激可用于经饮食调节、压力疏导和药物治疗等初始治疗无效的顽固性胃瘫患者。最近，内镜下幽门肌切开术（G-POEM）成为了一种治疗顽固性胃瘫的新的内镜技术。本研究报道了一组经胃电刺激治疗无效、后经透视引导下G-POEM补救治疗成功治愈的顽固性胃瘫病例。

方法：回顾性分析经胃电刺激治疗失败后接受G-POEM治疗的顽固性胃瘫患者的病历资料。所有G-POEM手术均在透视（了解胃电刺激器的安放位置）引导下进行。手术前后采用胃瘫主要症状指数（GCSI）和核素胃排空显像来评估胃动力。患者术后随访时间为1－18个月。

结果：5例顽固性胃瘫患者在胃电刺激治疗失败后接受G-POEM治疗。透视下可见胃电刺激器及其导丝位于胃的不同部位，其中1例胃电刺激器位于胃窦接近幽门肌切开处。5例手术均顺利完成，未出现任何并发症。术后1个月，患者GCSI平均下降62%；术后2个月，患者胃排空情况显著改善。

结论：对于胃电刺激治疗失败的顽固性胃瘫患者，G-POEM是一种安全有效的补充治疗措施，而且无需取出胃电刺激器。在幽门肌切开过程中，为了能观察到胃电刺激器的位置、避免切断胃电导线，G-POEM手术操作应在透视引导下进行。

关键词：胃瘫；内镜下幽门肌切开术；幽门肌切开术；胃电刺激；透视

### A comparison of APACHE II, BISAP, Ranson's score and modified CTSI in predicting the severity of acute pancreatitis based on the 2012 revised Atlanta Classification

### APACHE II、BISAP、Ranson 评分和改良CTSI对急性胰腺炎严重程度（基于Atlanta 2012分类）预测价值的比较

[摘要] 目的：本研究前瞻性比较了急性生理和慢性健康状况评分（APACHE Ⅱ）、急性胰腺炎严重程度床边指数（BISAP）、Ranson评分与改良CT严重度指数（CTSI）这四种方法，评估印度北部一家三甲医院急性胰腺炎患者疾病严重程度（基于Atlanta 2012分类）的准确性。

方法：前瞻性纳入2015年3月至2016年9月间我院收治的50例急性胰腺炎患者，对所有入组病例进行APACHE Ⅱ、BISAP和Ranson评分，并基于胰腺对比增加CT进行改良CTSI评估。基于受试者工作特征（ROC）曲线评估这些评分系统预测急性胰腺炎严重程度的最佳阈值和曲线下面积（AUC），并对这些评分系统的预测价值进行前瞻性比较。

结果：50例急性胰腺炎患者中14例被评估为重症急性胰腺炎。15例患者出现胰腺坏死，14例出现持续器官功能衰竭，14例需要收治重症监护

室（ICU）。改良CTSI对重症急性胰腺炎、胰腺坏死、器官衰竭、入住ICU的预测价值均为最高， 其AUC分别为0.919、0.993、0.893和0.993。APACHE Ⅱ对重症急性胰腺炎和器官衰竭的预测价值均仅次于改良CTSI， 其AUC分别为0.934和0.931。APACHE Ⅱ对胰腺坏死、器官衰竭、入住ICU的预测具有较高的敏感性（分别为93.33%、92.86%和92.31%）和阴性预测值（分别为96.15%、96.15%和95.83%）。

结论：APACHE Ⅱ是预测急性胰腺炎严重程度的一项有效的评估系统， 有助于筛选出病情危重、需要早期复苏和尽快转诊上级医院的急性胰腺炎患者, 尤其在一些医疗资源匮乏的发展中国家。

关键词：急性生理和慢性健康状况评分（APACHE Ⅱ）；急性胰腺炎严重程度床边指数（BISAP）；Ranson评分；改良CT严重度指数

### Screening for colorectal cancer in Tianhe, Guangzhou: results of combining fecal immunochemical tests and risk factors for selecting patients requiring colonoscopy

广州市天河区结直肠癌筛查：基于粪便免疫化学检测和危险因素初步筛选出需要行结肠镜检查者

[摘要] 目的：探讨联合粪便免疫化学检测（FIT）和高危因素问卷调查（HRFQ）初筛出需行结肠镜检查者的结直肠癌（CRC）筛查方案应用于中国的可行性。

方法：2015－2016年间， 我们对中国广州市天河区45岁以上居民进行了一项CRC筛查项目。所有筛查对象均接受FIT检测和HRFQ调查， 作为初筛。对于FIT阳性和/或HRFQ调查结果考虑存在高危风险的受检者, 我们推荐其行进一步结肠镜检查。

结果：共计10 074名居民纳入CRC筛查项目, 其中17.5%的受检者FIT阳性, 19.4%经HRFQ调查被认为具有高危风险。经推荐, 有773名受检者接受了进一步的结肠镜检查。该筛查方案对非腺瘤性息肉、低危腺瘤、高危腺瘤和CRC的总体阳性预测值分别为4.9%、11.4%、15.9%和1.6%。其中， FIT阳性结果对上述四种病变的阳性预测值分别为5.2%、15.9%、22.5%和2.5%, HRFQ阳性结果分别为4.1%、10.2%、14.3%和1.4%, FIT和HRFQ均为阳性结果的阳性预测值分别为4.5%、16.4%、23.7%和2.8%。

结论：我们的两步法CRC筛查策略（FIT和HRFQ阳性者行进一步结肠镜检查）可有效发现早期CRC。鉴于对CRC和腺瘤较的高检出率和阳性预测值， 该筛查方案可继续用于我们这项大规模CRC筛查项目的后续研究。

关键词：结直肠癌；筛查；粪便免疫化学检测；高危因素；结肠镜；阳性预测值

### Male gender is associated with an increased risk of anastomotic leak in rectal cancer patients after total mesorectal excision

男性直肠癌患者全直肠系膜切除术后吻合口漏风险增高

[摘要] 背景：患者性别对于直肠癌全直肠系膜切除术（TME）后吻合口漏形成的影响目前仍存在争议。本研究旨在评估性别与吻合口漏的相关性。

方法：回顾性分析2010－2014年间在我院接受TME手术且行一期吻合的直肠癌患者的临床资料。比较男性与女性患者术后吻合口漏发生率。

结果：入组的956例直肠癌患者中, 男性587例（61.4%）, 女性369例（38.6%）。相较于女性患者, 男性患者中吸烟和饮酒的比例更高, 但有腹部手术史者较少；术前肠梗阻的比例更高， 肿瘤本身也相对较大。全组病例中出现术后吻合口漏81例（8.5%）， 其中男性患者发生率高于女性患者（10.6% vs. 5.1%, $P = 0.003$）。多因素逻辑回归分析证实, 男性是术后吻合口漏发生的独立危险因素（OR = 2.41, 95% CI: 1.37－4.23, $P = 0.002$）；对于腹腔镜TME手术的亚组分析也得到了相同的结果（OR = 2.11, 95% CI: 1.15－3.89, $P = 0.016$）。

结论：男性直肠癌患者TME手术后吻合口漏的发生风险相对较高。保护性肠造口或可为吻合口提供保护, 降低吻合口漏的发生风险, 尤其是对于男性患者。

关键词：直肠癌；吻合口漏；性别；危险因素；全直肠系膜切除术；一期吻合

### Case report（病例报告）

### Locally infiltrative inflammatory fibroid polyp of the ileum: report of a case showing transmural proliferation

表现为透壁生长的局部侵袭性回肠炎性纤维样息肉

### Coeliac disease as a potential cause of idiopathic portal hypertension: a case report

乳糜泻是特发性门静脉高压症的潜在原因：一例报告

### Chronic hepatitis E – an emerging disease in an immunocompromised host

慢性戊型肝炎：免疫缺陷患者的一种新发疾病
